# Effects of plant species richness on ^13^C assimilate partitioning in artificial grasslands of different established ages

**DOI:** 10.1038/srep40307

**Published:** 2017-01-09

**Authors:** Longhua Xu, Buqing Yao, Wenying Wang, Fangping Wang, Huakun Zhou, Jianjun Shi, Xinquan Zhao

**Affiliations:** 1 Key Laboratory of Adaptation and Evolution of Plateau Biota, Northwest Plateau Institute of Biology, Chinese Academy of Sciences, Xinning Road 31, Xining 810008, China; 2 University of Chinese Academy of Sciences, Beijing 100049, China; 3 The Key Laboratory of Restoration Ecology in Cold Region of Qinghai Province, Xining, 810008, China; 4 College of Life Science and Geography, Qinghai Normal University, Wusi west 38, Xining 810008, China; 5 Qinghai Academy of Animal and Veterinary Sciences, Hutai A lane 3, Xining 810016, China

## Abstract

Artificial grasslands play a role in carbon storage on the Qinghai–Tibetan Plateau. The artificial grasslands exhibit decreased proportions of graminate and increased species richness with age. However, the effect of the graminate proportions and species richness on ecosystem C stocks in artificial grasslands have not been elucidated. We conducted an *in situ*^13^C pulse-labeling experiment in August 2012 using artificial grasslands that had been established for two years (2Y), five years (5Y), and twelve years (12Y). Each region was plowed fallow from severely degraded alpine meadow in the Qinghai-Tibetan Plateau. The 12Y grassland had moderate proportions of graminate and the highest species richness. This region showed more recovered ^13^C in soil and a longer mean residence time, which suggests species richness controls the ecosystem C stock. The loss rate of leaf-assimilated C of the graminate-dominant plant species *Elymus nutans* in artificial grasslands of different ages was lowest in the 12Y grassland, which also had the highest species richness. Thus the lower loss rate of leaf-assimilated C can be partially responsible for the larger ecosystem carbon stocks in the 12Y grassland. This finding is a novel mechanism for the effects of species richness on the increase in ecosystem functioning.

Grassland covers nearly one-fifth of the world’s land surface and approximately 24 million square kilometers^1^ of grassland soils store a large quantity of approximately 200–300 Pg C^2^. The total carbon storage in grassland in China is 44.09 Pg, which accounts for 9–16% of the total carbon storage in the world^3^,^4^,^5^. Alpine meadows are a moderate C sink^6^,^7^,^8^ and a large C pool because of their high productivity and low decomposition rate^9^. These features are caused by low temperatures in the growing season^10^,^11^. However, the conversion from carbon sink and source occurs following certain types of grassland management and changes in land use^12^,^13^,^14^.

The Qinghai–Tibetan Plateau has a highly sensitive and fragile ecosystem and grasslands cover 1.5 million Km^2^. This area represents 40% of the total grassland area in China^15^. A previous survey^16^,^17^ showed that there was 0.045 million Km^2^ of “black soil beach” (severely degraded grassland that cannot recover naturally) in the alpine meadow in the region that forms the source of three rivers. This land can be restored rapidly through the establishment of artificial or semi-artificial grassland. Artificial grasslands generally have low soil storage capacity compared to natural alpine meadow on the Qinghai–Tibetan Plateau, particularly for newly established grasslands with low species diversity and degraded grassland that is dominated by forbs^18^. There are currently 1.5 million Km^2^ of artificial perennial grassland, and these areas were established in different years and differ in their species diversity, community structure, and succession stages. Revealing the C dynamics of artificial grasslands of the Qinghai-Tibetan Plateau and the factors influencing their C storage capacity is crucial to understanding the regional and global C budget^19^. However, the carbon allocation patterns and determinations of artificial grasslands established for different lengths of time are poorly understood. Previous research has found ecosystem carbon storage was strongly influenced by plant diversity^20^,^21^. Plant diversity increases root biomass or root carbon concentration^22^,^23^,^24^ and microbial community activity^25^,^26^,^27^. Additionally, carbon storage increases with higher inputs of root carbon residues^28^ or with more plant-derived recalcitrant compounds^29^ and is directly influenced by the microbial community activity^21^,^30^,^31^,^32^,^33^,^34^.

Prior studies in the alpine meadows of the Qinghai–Tibetan Plateau have indicated that community structure determines the ecosystem carbon storage capacity^18^,^35^,^36^,^37^ and plant photo-assimilated carbon cycling^38^. Another study found the species richness and root-shoot ratio were the major controlling factors of soil C stocks when comparing the native alpine meadow with winter grazing, cultivated perennial and annual pastures^18^. The variations in community structures in these studies are all associated with changes in species richness and proportions of graminate. In artificial grassland on the Qinghai–Tibetan Plateau, graminate is typically the cultivated grass species, some of which could be replaced by forbs with increasing planting years and different anthropogenic management strategies. Graminate are fast-growing grasses that typically have strong nutrient-absorbing capacity and low below-ground allocation compared with other forbs in alpine meadow environments. However, the effects of the different graminate and of the species richness on the ecosystem C store of artificial grasslands of varying ages remains unclear. These differences may have significant effects on the restoration and management of degraded grassland.

In this study, we hypothesize that a low proportion of graminate grasses or high species richness would have positive effects on the C store in high-altitude artificial grasslands. We used an *in situ*^13^C pulse-labeling experiment (plant diversity has pronounced effects on the enhanced accumulation of recently fixed carbon but not existing carbon^21^) to test these hypotheses on three general grasslands that were ploughed from “black beach” for two years (2Y, young), five years (5Y, middle-aged) and twelve years (12Y, old). The purposes of this study were the following: (1) to quantify the partitioning of recently fixed carbon among shoot, root, and soil and fluxes in the plant-soil system and (2) to estimate the effects of graminate grasses and species richness of artificial grasslands on carbon cycling in the plant-soil system.

## Results

### Total carbon stocks and biomass

Biomass, root-shoot ratio, species richness and above and below-ground total carbon stocks for different established ages of artificial grassland are shown in Table 1. Artificial grassland after 5 years had the least above-ground biomass, and the 12-year grassland had the most. The total carbon showed no significant differences in either above- or below-ground biomass for the three types of grassland. The species richness is 9.4, 2.6, and 15.5 for 2Y, 5Y and 12Y grasslands, respectively.

### ^13^C dynamics and partitioning in the plant–soil system

**Figure 1:**
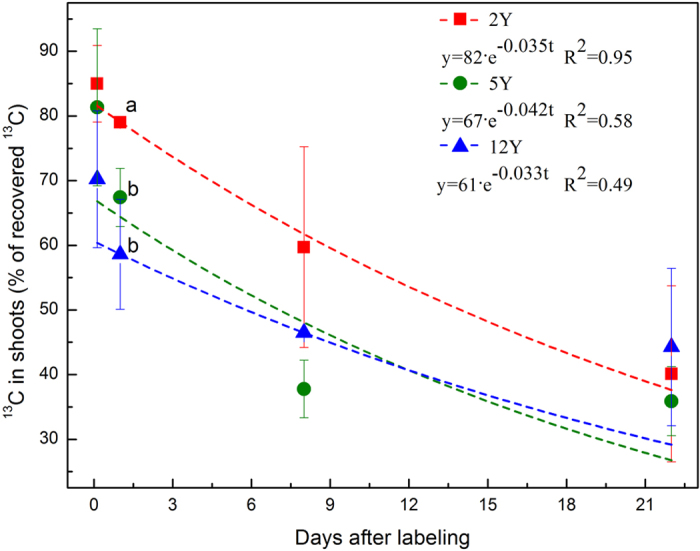
^13^C dynamics in shoots during the chase period. 2Y, planting of artificial grass for two years; 5Y, planting of artificial grass for five years; 12Y, planting of artificial grass for twelve years. Data are shown as the mean ± standard deviation (n = 3, *P* < 0.05). Different letters indicate significant differences among the three general types of lands at each sampling date.

After the labeling, ^13^C in shoots of the three general types of artificial grasslands followed an exponential decrease during the chase period (Fig. 1). The decline reflects the newly assimilated ^13^C allocated to below-ground stocks and the C loss by respiration. The dynamics of ^13^C allocation in shoots were similar for three general types of artificial grasslands, but the recovery of ^13^C in shoots differs significantly between the 2Y and 12Y artificial grasslands after the 1st day of labeling.

**Figure 2:**
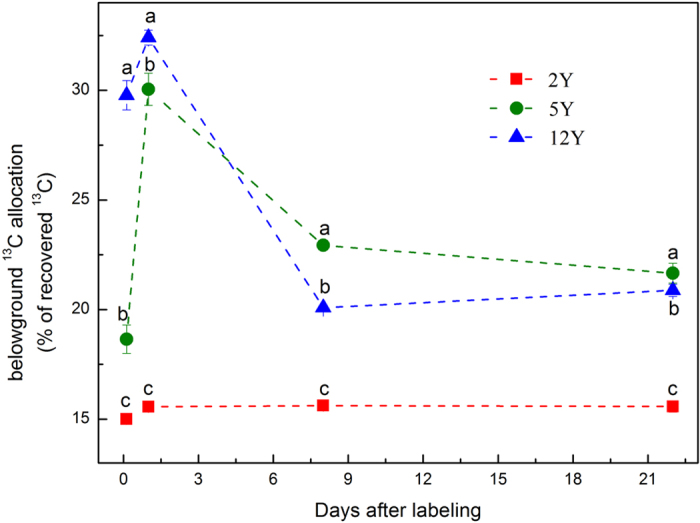
^13^C allocation to below-ground C pools during 22 days after labeling.

**Figure 3:**
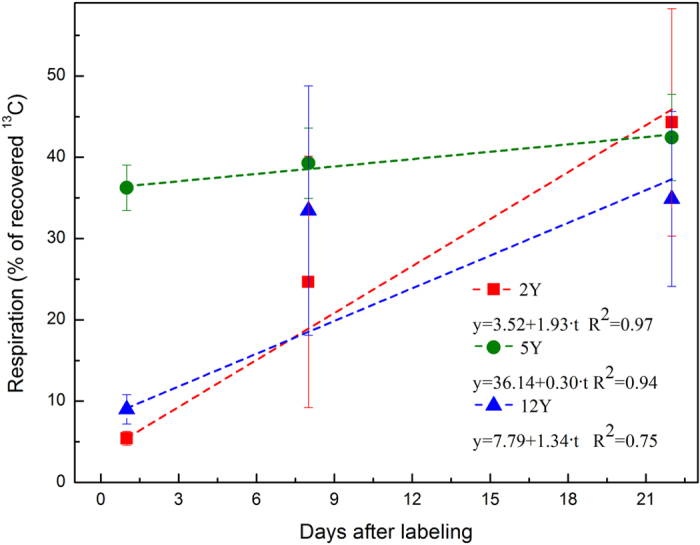
^13^C losses by respiration during 22 days after labeling.

The newly assimilated ^13^C allocated from shoots to below-ground C pools and to respiration between 3 h and 22 days after labeling amounted to 44.9, 45.5, and 26.0% of recovered ^13^C in the grasslands aged 2Y, 5Y, and 12Y, respectively (Fig. 1). Different sections of the sites were used for below-ground C allocation (Fig. 2) and for respiration (Fig. 3). The amount of ^13^C transferred to below-ground pools was greater in the 12Y and 5Y sites than in the 2Y site, and differences were significant among 12Y, 5Y and 2Y grasslands at all sampling times (Fig. 2). The maximum recovery of ^13^C in below-ground pools occurred on the 1st day after the labeling in the 12Y site. Carbon loss by respiration for the 5Y site was greater on the 1st day and the 8th day of the chase period (Fig. 3), and the maximum loss of ^13^C by respiration was not significant at 22 days, the end of the labeling time.

**Figure 4:**
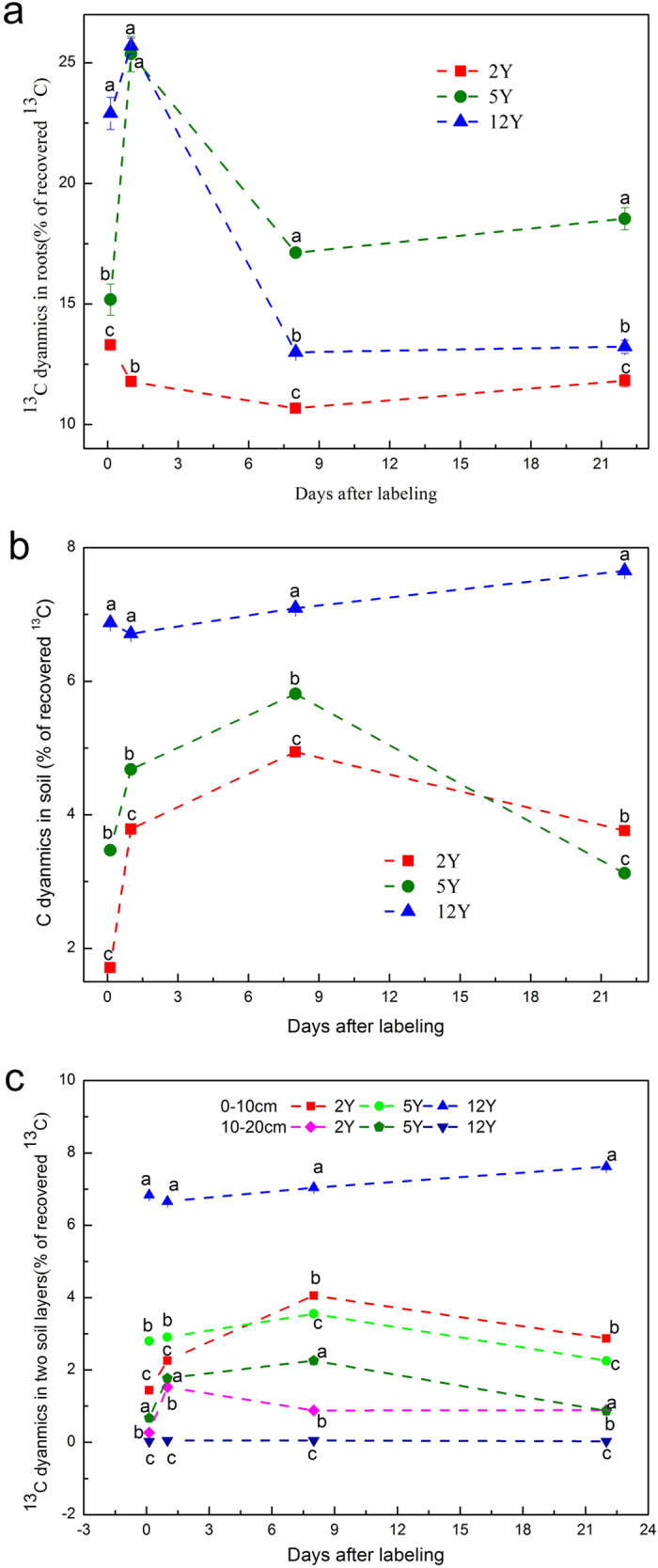
^13^C dynamics during 22 days in roots (**a**) and soil (**b**) (0–20 cm) and in two layers of soil (**c**) (0–10 cm, 10–20 cm).

On the 1st day after labeling, assimilated ^13^C in roots for the 2Y, 5Y, and 12Y plots was 11.8, 15.3, and 25.7%, respectively (Fig. 4a). The recovery of ^13^C in roots showed significant differences for all sampling dates during the labeling period. During the following days, the percentage of ^13^C for 5Y grassland gradually increased to 18.5% at 22 days, whereas the percentage for 12Y grassland decreased to 13.2%, and in the 2Y plots, the percentage remained constant at 11.8% (Fig. 4a).

At 3 hours after labeling, 1.7%, 3.5%, and 6.9% of the recovered ^13^C was found in the soil of the 2Y, 5Y, and 12Y artificial grassland, respectively. The ^13^C recovery in the 12Y site was much higher than in the other two grasslands (Fig. 4b), with a larger percentage of recovered ^13^C located in the topsoil at a depth of 0–10 cm for the 12Y plots (Fig. 4c). The maximum recovery of ^13^C in soil was 8 days after the labeling in the 2Y (4.9%) and 5Y (5.8%) plots (Fig. 4b), and significant differences were observed among the three grasslands at all sampling times (Fig. 4c).

**Figure 5:**
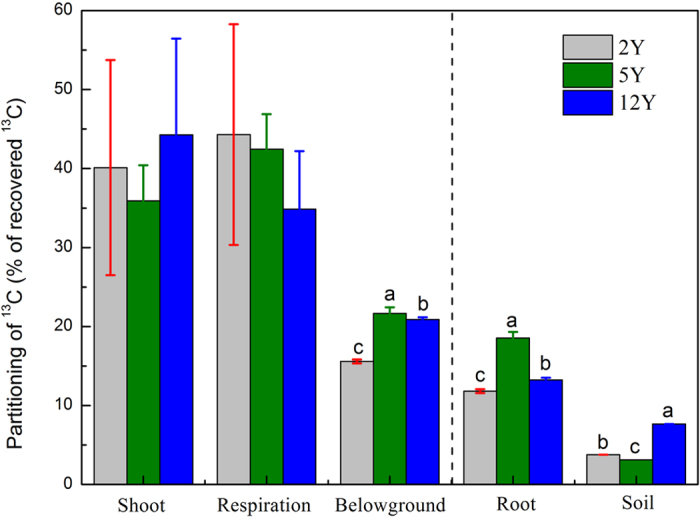
Partitioning of ^13^C 22 days after the assimilation.

At the end of the chase period, 15.6%, 21.7% and 20.9% of recovered ^13^C in the 2Y, 5Y, and 12Y grasslands, respectively, had been transferred to below-ground C pools (Fig. 5). At 22 days after the labeling, 44.3% and 42.5% of the recovered ^13^C was used for respiration in the 2Y and 5Y grasslands, respectively, more than in the 12Y site (34.9% of recovered ^13^C) (Fig. 5). Within the below-ground C pools, the smallest proportion of ^13^C was incorporated into the soil in all three types of grasslands (Fig. 5). At 22 days after labeling, 18.5% of recovered ^13^C was stored in roots in the 5Y plot, more than in the other two ages of grasslands (11.8% in 2Y and 13.2% in 12Y).

### Mean residence time

Mean residence time (MRT) of net assimilated C was determined to evaluate its lifetime in the plant-soil system. The longest MRT detected was 51.3 days in 12Y, followed by 39.8 days in 5Y and 37.9 days in 2Y.

### Relationships between ratio (graminate biomass/forb biomass) and recovered ^13^C in soil and MRT

Relationships between ratio (graminate biomass/forb biomass) and recovered ^13^C in soil and MRT were not significant in linear fittings. The quadratic curve fittings (% Soil_-rec_^13^C = −0.99 × ratio^2^ + 9.44 × ratio-12.61, and MRT = −2.71 × ratio^2^ + 25.34 × ratio-2.85) showed that the peak value of recovered ^13^C in soil of artificial grassland was 9.89% when the ratio value reached 4.77, and the peak value of MRT was 56.46 when the ratio value was 4.68.

### Species foliage ^13^C

**Figure 6:**
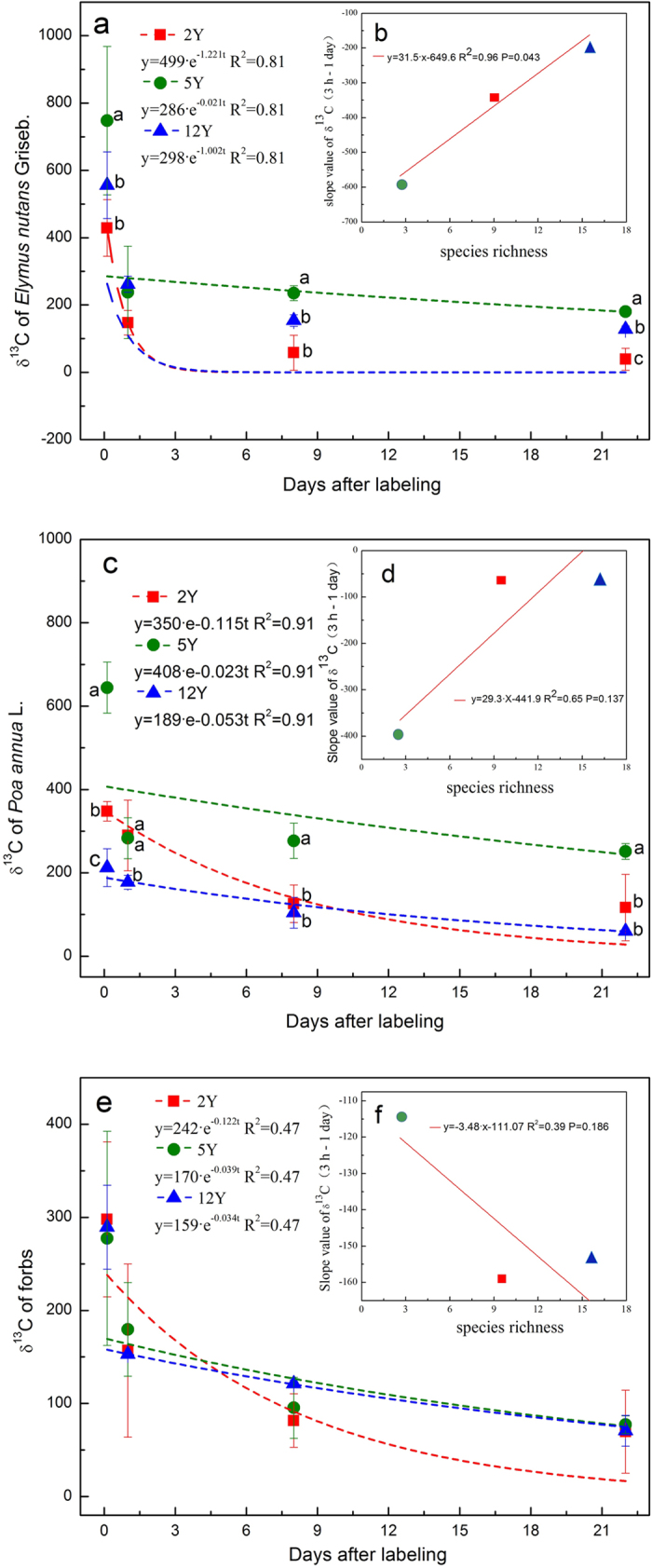
^13^C in foliages of three species in the three ages of grassland. (**a**) δ^13^C in foliages of *Elymus nutans* Griseb.; (**b**) relationship between slope values of δ^13^C(3h-1d) and species richness of *E. nutans* in the three grasslands; (**c**) δ^13^C in foliages of *Poa* annua; (**d**) relationship between slope values of δ^13^C(3h-1d) and species richness of *P.* annua in the three grasslands; (**e**) δ^13^C in foliages of forbs; (**f**) relationship between slope values of δ^13^C(3h-1d) and species richness of forbs.

The ^13^C values decreased in the foliage of three species with increases in the labeling time (Fig. 6). For *E. nutans* and *Poa annua*, the ^13^C values were greater in 5Y than in the other two grasslands at all labeling times except for the first day (*P* < 0.05) (Fig. 6a,c). The largest slope value of ^13^C for *E. nutans* was observed in the 12Y plot from 3 h to one day after labeling (Fig. 6b), and the smallest slope value was in the 5Y plot (Fig. 6b), with similar results for *P. annua* (Fig. 6d). The relationship between slope values of ^13^C from 3 h to one day for *E. nutans* and species richnesses of the three artificial grasslands was significant in linear fittings (Fig. 6b). However, the largest slope value of ^13^C for forbs was observed in the 5Y plot (Fig. 6f) and the relationship between slope values of ^13^C from 3 h to one day and species richnesses of the three artificial grasslands was not significant in linear fitting (Fig. 6f).

## Discussion

Different land uses have significant effects on the exchange and sequestration of ecosystem C and the percentages of C allocated to root pools and soil pools differed significantly. We also observed the different C residence time for different age artificial grasslands.

Assimilated ^13^C was recovered in shoots (70–85%), roots (13.3–22.9%), and soil (1.7–6.9%) at 3 h after labeling in all land types (Figs 1 and 4). Loss or export of recently fixed C of shoots was 20.4%, 31.3%, and 38.8% in the first 24 h for 2Y, 5Y, and 12Y, respectively, all of which are much lower than the value of 77% reported after two sequential pulse-labelings^39^. The maximum decrease rate in leaf occurred between 0 h and 24 h after labeling (Fig. 1), and the maximum translocation rate to below-ground pools occurred within 24 h after labeling (Fig. 2). This result is similar to those of studies reported earlier^18^,^40^,^41^. Loss through respiration, which increased between 1d and 22d in the 2Y and 12Y sites (Fig. 2) but remain unchanged in the 5Y site (Fig. 2), is consistent with the grazed plot studied by Hafner *et al*.^19^. However, this parameter showed no significant changes for the three plots during the labeling period.

Roots are considered the major C sink within the below-ground pools^40^. The results of the experiment indicated that more of the below-ground carbon was contained in roots than soil pools (Figs 4 and 5). The artificial grasslands in our study contained perennial species such as herbage plants and forbs and had developed a relatively good rooting system that can be used as carbon storage. More than 70% of the allocated ^13^C in soil was in the top 10 cm of soil (Fig. 4c), due to the superficial root biomass allocation in artificial grassland compared with natural alpine meadow.

Significant differences were found in the partitioning of ^13^C into below-ground C pools among the 2Y, 5Y, and 12Y grasslands. For 5Y and 12Y, there was a significantly greater percentage of assimilated ^13^C in below-ground C pools (12Y, 21.7%; 5Y, 20.9%), than for 2Y (15.6%) (Figs 2 and 5). The partitioning percentage was lower than in previous pulse-labeling studies, 30–50% reported by Kuzyakov *et al*.^42^, 58.7% reported by Wu *et al*.^40^, 40% reported by Hafner *et al*.^19^, 61% by Zou *et al*.^38^ and 22–43% by Zhao *et al*.^18^. This may be because of the lower species richness (Table 1) and simpler community structures of artificial grasslands than in the aforementioned studies^18^,^38^,^40^. The ecological structure of artificial grasslands is not natural, and it is potentially unstable and fragile.

Artificial grasslands of different established ages showed different capacities for recovery of soil ^13^C stock. The proportion of carbon remaining in the soil was higher in the 12Y plot (7.65%) than in the 2Y (3.76%) and 5Y (3.12%) plots (Figs 4 and 5); significantly, the values for the 2Y and 5Y plots were similar to the 3% reported in perennial *E. nutans* grassland studied by Zhao *et al*.^18^, but all three values were lower than reports from native meadow^18^,^19^,^38^. This was not controlled by changes in the proportion of graminate because our result showed that a moderate proportion of graminate would have the highest value of recovered ^13^C. The 12Y plot had the highest species richness (15.5 ± 1.1) among the three artificial grasslands, which may explain this condition. According to the complementary niche hypothesis, higher species richness will benefit ecosystem function^43^. One of the reasons might be the high below-ground biomass for the 12Y site (Table 1). Many studies have shown that carbon allocation within the subsurface pool changed in response to species richness^14^,^44^. Moreover, coexisting species under conditions of high species richness may occupy different niches in the community, which improves the utilization efficiency and the competition for limited resources in soils^14^, so the plant roots of communities with higher species richness may produce more exudates, increase rhizodeposition and decompose faster, enhancing soil organic matter turnover^45^,^46^,^47^. In addition, our results revealed that the artificial grassland established for 12 years was progressing slowly toward the succession of natural grassland and had a more stable community structure than grasslands established for 2 or 5 years.

The MRT of ecosystem C in the 12Y grassland was 1.4 times longer than the 2Y. This implies that the photosynthetic C stocked in the 12Y has a lower rate of loss than that do other artificial grasslands. This finding was not caused by the proportion of graminate because the longest MRT was associated with moderate graminate proportions and not low graminate proportions. We found that the C transfer and loss rates in the 12Y grassland with higher species richness were lower (shallow slopes in Fig. 6) in the leaves of the graminate plants *E. nutans* and *P. annua* by labeling from 3 h to one day than those in the 2Y and 5Y with lower species richnesses. The lower loss rate of leaf-assimilated C can partially explain the larger ecosystem carbon stocks in the 12Y grassland. This result indicates the effect of species richness on system C stock is derived not only from diversified functions of increased species and from intrinsic changes in the functions of the dominant original species. Therefore, the transfer and loss of assimilated C in the plant aboveground material was also affected by biodiversity, though the majority of existing studies focused on the aboveground litter decomposition^48^,^49^ and the below-ground microbial biomass and activity^50^,^51^,^52^.

One of the possible explanations that account for this lower loss rate of leaf-assimilated C is associated with decreased aboveground respiration in the 12Y. Previous studies reported that higher plant diversity mitigated C losses^53^,^54^, and a recent study demonstrated lower aboveground respiration for the perennial artificial grass (with higher biodiversity) than for the annual artificial grass (with lower biodiversity) on the Qinghai–Tibetan Plateau^18^. Alternatively, in all three artificial grasslands, *E. nutans* and *P. annua* were two of the fast-growing plant species, and these grasses were shown to have small root-shoot ratios. The reduced rates of transfer and loss of leaf assimilated C might derive from an adaptive resource allocation response to increased aboveground competition in the 12Y compared with the other artificial grasslands.

## Materials and Methods

### Site description

The study was conducted at a ranch in Maqin County, of the Golok Tibetan Autonomous Prefecture of Qinghai Province, China (34° 20′–22′ N, 100° 30′–29′ E) at an altitude of 4,120 m. The station has a plateau continental climate characterized by strong solar radiation with long, cold winters and short, cool summers. The annual average temperature is −0.6 °C, ranging from −10 °C in January to 11.7 °C in July. The mean annual precipitation is 513 mm, occurring mainly in the short summer from May to September. There is no entirely frost-free period. The natural vegetation type of the research area is alpine meadow, most of which has been degraded by human disturbance (overgrazing and fertilizer use) and climate change. The artificial grasslands were revegetated in the most severely degraded alpine meadows (often termed “black beach” or “black-soil land”), with mixed sowing of native perennial grasses (mixed *E. nutans.* and *P. annua* with the same proportion) in recent years.

In this study, we selected three 100 × 100 m sites that were artificially planted and restored from “black-soil land” and that have been growing for different lengths of time: two years (2Y), five years (5Y) and twelve years (12Y). All of the sites have been used as winter pasture by local herdsmen. All of the plant species were perennials. The species composition of 2Y was *E. nutans, P. annua, Festuca sinensis*, and the forbs mainly included *Pedicularis kansuensis, Potentilla nivea, Gueldenstaedtia diversifolia, Viola yedoensis, Parnassia trinervis, Leontopodium nanum*, and *Oxytropis ochrocephala*. The species composition of 5Y was *E. nutans, P. annua,* and the forbs mainly included *P. nivea, P. trinervis*. The species composition of 12Y included *E. nutans, P. annua, F. sinensis, Stipa aliena.* The forbs mainly included the following: *P. kansuensis, Saussurea superb, P. nivea, G. diversifolia, V. yedoensis, P. trinervis, L. nanum, Gentiana straminea, Lancea tibetica, Ajania tenuifolia, Polygonum sibiricum* and *O. ochrocephala*. The ratios of graminate to forbs were 7.3, 2.2, and 3.3 for the 2Y, 5Y and 12Y plots, respectively. The proportions of the different forbs varied. During the experimental periods, most plant species were at the developmental stages of flowering or fruiting. The rainfall was plentiful, and plants in all three types of artificial grasslands grew well without drought stress during our experiment. More details are provided in Table 1.

### Pulse labeling

We randomly established three replicate plots (1 × 1 m, 50 cm height) in each artificial grassland. The closest distance between two neighboring plots was approximately 5 m. We installed a wire skeleton in each plot in the late afternoon of the day before isotope labeling and covered it with a transparent polyethylene film just before labeling (film with more than 96% transmittance of photosynthetically active radiation). Each chamber was composed of wire skeleton and polyethylene film. We inserted the chambers into the soil to a depth of approximately 5 cm and packed them with a nylon mesh with pore size of 45 mm. These meshes were extended to 10 cm depth to cut off roots from plants outside of the labeling chambers, while allowing nutrient and water exchange between the soil inside and outside of the chambers. Extra-fine earth was packed firmly around the base of the chamber to reduce gas leakage. The inner surface of the chamber was smeared with anti-fog agent to reduce water vapor condensation during labeling, which can increase light intensity and reduce the ^13^CO_2_ dissolved into water drops on the chamber’s inner surfaces.

Pulse labeling was carried out at 11:00 in the morning on August 1, 2011, which was a clear day. Labeling began immediately after the polyethylene film was closed and tightly sealed. A beaker containing a solution of 40 mL distilled water and 8.0 g Na_2_^13^CO_3_ (with ^13^C to 99 at.%) was placed in the center of the chamber in each plot, the ^13^CO_2_ was released by careful injection of 28.4 mL of 5 mol/L H_2_SO_4_ into the beaker, and the chamber was then immediately sealed. The volume of ^13^CO_2_ released from the chemical reaction was 1.66 L at least. It would decrease quickly to a low level according to reported photosynthetic rate at both leaf and community levels^55^, so the total volume of released ^13^CO_2_ did small change in air pressure inside the chambers. The air in each chamber was circulated by an electric fan (12 V, 0.21 A) in the center of each chamber to guarantee a uniform air environment. The polyethylene film of the chambers was beaten gently by hand to remove the condensed water vapor, and the polyethylene film was removed after 3 hours. The flow rate and labeling time were determined according to photosynthetic rates and below-ground productivity as previously measured^18^,^35^,^38^,^40^, to ensure sufficiently higher ^13^C abundances in both shoot and root samples, compared with unlabeled control samples, during the whole chase period. The CO_2_ concentration in the chambers was not measured during labeling. It can be expected to decrease quickly to a low level according to reported photosynthetic rates at both leaf and community levels^6^,^55^,^56^.

### Sample collection

Shoot samples were collected 3 h and 1, 8 and 22 days after labeling. Above-ground plant parts of all species were harvested and pooled as shoot samples by clipping at the soil surface before the soil was sampled. Foliage samples of different species were collected separately. Only green shoots were used for further ^13^C analysis. Soil cores of 5 cm in diameter were taken to 20 cm depth immediately and at 3 h, 1, 8, and 22 days after labeling. Soil and root samples were taken from two layers: 0–10 cm and 10–20 cm. All roots and soil in the cores were carefully extracted and sieved with a 2 mm screen. The soil samples that passed through the sieve were air-dried and stored at 4 °C before being analyzed for total carbon and ^13^C. The sampled roots were carefully washed by wet sieving though a 0.5 mm screen to remove attached soil and dark-brown/black debris. The roots were further separated into living and dead components based on their color. The shoot and root samples were oven-dried at 105 °C for 48 h. Only data from living roots are mentioned in this work^40^.

### Measurement and calculations

To remove all carbonates, soil samples were prepared by washing in 0.1 mol/L HCl until no air bubbles appeared. The acid-treated samples were oven-dried at 105 °C for 24 h. They were then ground manually with a pestle and mortar to homogeneously fine powders. Shoot and root samples were also ground using a MM 200 steel ball mill (Retsch GmbH, Haan, Germany). Carbon contents and the ^13^C/^12^C ratio in the samples were measured with a MAT 253 stable isotope ratio mass spectrometer system (Thermo Fisher Scientific Inc., Bremen, Germany).

The natural abundance of ^13^C in samples is expressed as ^13^C % units relative to Pee Dee Belemnite. To facilitate comparisons with other studies, we also calculated the enrichment values as ^13^C (at%) excess, the increase in ^13^C atoms due to pulse labeling expressed as the percentage of total carbon atoms in the sample, using the following equations:


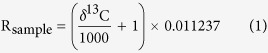



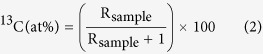



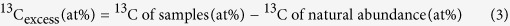


where R sample is the isotope ratio (^13^C/^12^C) of the sample, and 0.011237 is the ratio of ^13^C/^12^C in Pee Dee Belemnite. ^13^C(at%) represents the percentage of ^13^C atoms among the total carbon atoms. To estimate the amount of ^13^C incorporated into various plant and soil pools, the following equation was used:


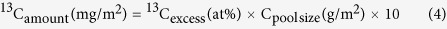


where carbon pool size is the carbon content in shoots, roots and soil and was assumed to be constant during the whole chase period. Atmospheric background was corrected using ^13^C(at%) excess instead of ^13^C as in the above equation.


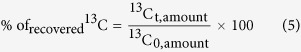


Here, ^13^C_0,_ represents the weight (mg m^−2^) of ^13^C in the pool 3 hours after the labeling.

Ecosystem respiration was calculated according to the following equation:


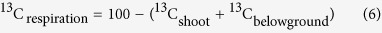


where ^13^C respiration is the % of recovered ^13^C of ecosystem respiration, and ^13^Cshoot is the % of recovered ^13^C in shoots ^13^C_belowground_ is the % of recovered ^13^C in the below-ground C pool.

Mean residence time is the average time a C atom remains in a compartment and is defined as the ratio of the holding capacity (pool size) and (net) C flux through the pool^35^. MRT was determined by Eqs 7 and 8 57:


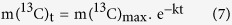


where m(^13^C)_t_ is the mass (in mg) of C present in the plant–soil compartment at t time, m(^13^C)_max_ is the amount of ^13^C at the peak, and t is the time after labeling:


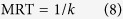


### Statistical analysis

Normality of aboveground and below-ground plant biomass and SOC stocks was tested using the Kolmogorov–Smirnov test. The significance of differences among the three treatments considering the aboveground and below-ground plant biomass and ^13^C stocks was tested by ANOVA, which was calculated separately for each layer; *P* < 0.05 was considered statistically significant for treatment means. We used nonlinear least squares (function Bnls^) to fit Eq. 5. Statistical analysis was performed using SAS 9.2 for Windows.

## Additional Information

**How to cite this article**: Xu, L. *et al*. Effects of plant species richness on ^13^C assimilate partitioning in artificial grasslands of different established ages. *Sci. Rep.* **7**, 40307; doi: 10.1038/srep40307 (2017).

**Publisher's note:** Springer Nature remains neutral with regard to jurisdictional claims in published maps and institutional affiliations.

## Figures and Tables

**Table 1:** Biomass, root-shoot ratio, total carbon, species richness and ratio (graminate biomass/forb biomass) for the three types of grasslands.

Treatment	Depth	2Y	5Y	12Y
Above-ground biomass (g m^−2^)		203.9 ± 9.3b	135.4 ± 7.6c	261.2 ± 15.5a
Belowground biomass (g m^−2^)		919.1 ± 25.0b	1058.4 ± 162.5b	1733.1 ± 225.8a
Root-shoot ratio		4.48 ± 0.74 ns	7.92 ± 0.58 ns	7.17 ± 1.82 ns
Shoot total carbon (mg g^−1^)		428.3 ± 24.2 ns	421.6 ± 33.7 ns	414.6 ± 9.8 ns
Root total carbon (mg g^−1^)	0–10 cm	404.9 ± 45.0 ns	428.7 ± 33.9 ns	400.2 ± 20.9 ns
	10–20 cm	352.3 ± 44.7 ns	400.7 ± 15.1 ns	353.8 ± 24.2 ns
Soil total carbon (mg g^−1^)	0–10 cm	28.7 ± 7.5 ns	33.2 ± 20.4 ns	41.6 ± 9.2 ns
	10–20 cm	43.4 ± 19.4 ns	54.9 ± 26.8 ns	41.4 ± 19.0 ns
Species richness		9.4 ± 1.4b	2.6 ± 0.4c	15.5 ± 1.1a
Ratio (graminate biomass/forb biomass)		7.3	2.2	3.3

2Y, planting of artificial grass for two years; 5Y, planting of artificial grass for five years; 12Y, planting of artificial grass for twelve years. Different letters indicate significant differences among the three general types of lands (n = 3, *P* < 0.05); ns represents non-significant differences.
